# Investigation of Sulfonated Graphene Oxide as the Base Material for Novel Proton Exchange Membranes

**DOI:** 10.3390/molecules27051507

**Published:** 2022-02-23

**Authors:** Andrea Basso Peressut, Matteo Di Virgilio, Antonella Bombino, Saverio Latorrata, Esa Muurinen, Riitta L. Keiski, Giovanni Dotelli

**Affiliations:** 1 Department of Chemistry, Materials and Chemical Engineering “Giulio Natta”, Politecnico di Milano, Piazza Leonardo da Vinci 32, 20133 Milano, Italy; andreastefano.basso@polimi.it (A.B.P.); antonella.bombino@mail.polimi.it (A.B.); saverio.latorrata@polimi.it (S.L.); 2 Environmental and Chemical Engineering Research Unit, Faculty of Technology, University of Oulu, Pentti Kaiteran katu 1, FI-90014 Oulu, Finland; esa.muurinen@oulu.fi (E.M.); riitta.keiski@oulu.fi (R.L.K.)

**Keywords:** self-assembling membranes, graphene oxide, sulfonation, proton conductors, XRD, IEC

## Abstract

This work deals with the development of graphene oxide (GO)-based self-assembling membranes as possible innovative proton conductors to be used in polymer electrolyte membrane fuel cells (PEMFCs). Nowadays, the most adopted electrolyte is Chemours’ Nafion; however, it reveals significant deficiencies such as strong dehydration at high temperature and low humidity, which drastically reduces its proton conductivity. The presence of oxygenated moieties in the GO framework makes it suitable for functionalization, which is required to enhance the promising, but insufficient, proton-carrying features of GO. In this study, sulfonic acid groups (–SO_3_H) that should favor proton transport were introduced in the membrane structure via a reaction between GO and concentrated sulfuric acid. Six acid-to-GO molar ratios were adopted in the synthesis procedure, giving rise to final products with different sulfonation degrees. All the prepared samples were characterized by means of TGA, ATR-FTIR and Raman spectroscopy, temperature-dependent XRD, SEM and EDX, which pointed out morphological and microstructural changes resulting from the functionalization stage, confirming its effectiveness. Regarding functional features, electrochemical impedance spectroscopy (EIS) as well as measurements of ion exchange capacity (IEC) were carried out to describe the behavior of the various samples, with pristine GO and commercial Nafion^®^ 212 used as reference. EIS tests were performed at five different temperatures (20, 40, 60, 80 and 100 °C) under high (95%) and medium (42%) relative humidity conditions. Compared to both GO and Nafion^®^ 212, the sulfonated specimens demonstrate an increase in the number of ion-carrying groups, as proved by both IEC and EIS tests, which reveal the enhanced proton conductivity of these novel membranes. Specifically, an acid-to-GO molar ratio of 10 produces a six-fold improvement of IEC (4.23 meq g^−1^) with respect to pure GO (0.76 meq g^−1^), while a maximum eight-fold improvement (5.72 meq g^−1^) is achieved in SGO-15.

## 1. Introduction

Nowadays, graphene oxide (GO) is considered among the most attractive graphene-related materials. It can be described with good approximation as a one-atom-thick two-dimensional sheet of carbon atoms arranged in a texture similar to a honeycomb. The carbon atoms can be sp^2^- or sp^3^-hybridized and form chemical bonds with oxygenated functionalities. The most important are hydroxyl (–OH), epoxy (C–O–C) and carbonyl (C=O) groups, which are mainly located on the basal plane, and carboxyl (–COOH) and phenolate (–OPh) ones, whose steric hindrance compels their positioning at the sheet edges [1,2,3]. The random distribution of these moieties causes a nonstoichiometric atomic composition; therefore, a univocal molecular structure of GO is still under debate inasmuch as several preparation factors can directly influence it, such as the graphite source, the oxidizing agent and the reaction conditions. However, the presence of oxygenated functionalities endows GO with an amphiphilic nature that explains its dispersibility in aqueous media. The compresence of π-conjugated hydrophobic and aliphatic hydrophilic domains is one of the main reasons for the interesting self-assembling ability of this carbon-based material, which can stack to form large and thin free-standing paper-like membranes [4]. Other fascinating physicochemical properties derive from such a configuration, i.e., high mechanical strength, high acidity, variable electrical conductivity depending on the oxidation degree and intrinsic chemical reactivity [3,4,5]. The versatility of GO is in fact exploited in a wide range of applications, such as wastewater treatment [6,7], acid catalysis [8], power generation [9] and supercapacitors [10].

As a consequence of its high surface reactivity, GO structure can be easily tuned by performing an alteration of the oxygenated groups content or a convenient functionalization [2,4]. The purpose is the obtainment of derivatives with properties able to fulfill the specifications of those devices in which pure GO would not be sufficiently suitable. Reduced graphene oxide (rGO) and sulfonated graphene oxide (SGO) represent a clear example of such modifications. The former is prepared via a thermal or chemical reduction with the intent of increasing the C-to-O ratio up to values approaching pristine graphene [5], and finding employment in Li-ion batteries [11] and filters for water softening [6]. The latter is characterized by sulfonic acid groups grafted on the GO surface, which bestow a high ionic conductivity thanks to the facilitated solvation of movable protons [12], and it is mainly applied in the field of fuel cells [13,14,15,16,17,18].

Several authors have focused on researching an adequate sulfonation method to prepare SGO for fuel cell-related applications. Its typical employment is as an addition to electrolytes with the aim of enhancing water retention, proton conduction and mechanical properties. Zarrin et al. [15] studied SGO/Nafion^®^ nanocomposites as a potential replacement of unmodified Nafion^®^ in polymer electrolyte membrane fuel cells working at high temperature. They functionalized GO by reacting it with 3-mercaptopropyl trimethoxysilane (MPTMS) in toluene at 110 °C for 24 h and then oxidizing the intermediate at 25 °C for 24 h with a 30 wt.% solution of hydrogen peroxide (H_2_O_2_). The composite membranes, fabricated via the solution casting, demonstrated a significant enhancement in proton conductivity with respect to untreated Nafion^®^. The same material was proposed by Chien et al. [16] for high-performance direct methanol fuel cells. However, they employed a different precursor to prepare SGO, that is, sulfanilic acid. They added GO to a 0.06 M solution of sulfanilic acid at 70 °C, then dripped a 0.006 M sodium nitrite (NaNO_2_) solution under continuous stirring, maintaining the mixture at 70 °C for 12 h. The resulting SGO/Nafion^®^ proton exchange membranes, manufactured by bar coating, displayed a low methanol crossover and high proton conductivity at a low relative humidity. Li et al. [17] analyzed a sulfonated holey graphene oxide paper sandwiched between two sulfonated poly(ether ether ketone) (SPEEK) membranes. SGO was prepared by the combination of GO with an arenediazonium salt synthetized from sulfanilic acid, hydrochloric acid (HCl), NaNO_2_ and sodium hydroxide (NaOH). The performance of the combined materials was satisfactory from the point of view of proton exchange. Vinothkannan et al. [18] evaluated the practicability of SGO-Fe_3_O_4_-filled Nafion^®^ in proton exchange membrane fuel cells working at low humidity. SGO was obtained via a reaction with an aryldiazonium salt of sulfanilic acid in an ice bath, whereas the composite membranes were produced via solution casting. They observed the positive influence of SGO on the functional properties of Nafion^®^, stating the possibility of implementation in real devices. Despite such premises, the danger of some reactants required for SGO preparation and the difficulty to free them from the coupling with a polymeric matrix are still unsolved issues.

This work concerns the preparation and detailed study of self-assembling sulfonated graphene oxide (SGO-X) membranes as a potential base material for novel proton conductors to be employed in proton exchange membrane fuel cells (PEMFCs). The core of the procedure is the controlled reaction between GO and concentrated sulfuric acid aimed at decorating the GO framework with sulfonic acid groups (–SO_3_H), thereby favoring proton transport. Six acid-to-GO molar ratios (X = 1, 2.5, 5, 10, 15, 20) were explored to comprehend the effects of such a procedure on the morphology and properties of a GO structure. The corresponding SGO-X samples were extensively characterized via a thermogravimetric analysis (TGA), attenuated total reflection Fourier transform infrared (ATR-FTIR) and Raman spectroscopies, temperature-dependent X-ray diffraction (XRD), scanning electron microscopy (SEM) and energy-dispersive X-ray (EDX) spectroscopy. Furthermore, functional properties were preliminarily evaluated by means of ex situ ion exchange capacity (IEC) and electrochemical impedance spectroscopy (EIS) tests, in order to assess the suitability of this material for possible in situ experiments. A comparison was conducted with pure GO and Nafion^®^ 212, which was taken as an example of the industry-leading proton conductors’ family, with the aim of determining the potentiality of SGO-X membranes as innovative materials for the electrochemical energy generation sector.

## 2. Materials and Methods

### 2.1. Materials

Graphene oxide was purchased from Graphenea, Inc. (Cambridge, MA, USA) as a 0.4 wt.% aqueous dispersion with average particle size of less than 10 µm and a pH between 2.2 and 2.5. Starting from an elemental analysis performed by the vendor [19], the authors extrapolated an empirical GO formula (C_1.5_H_0.2_N_0.01_S_0.03_O) by exploiting the oxygen atoms content as normalization factor [14]. The associated molecular mass of 35.3 g mol^−1^ was compatible with literature values [20,21] and preliminary energy dispersive X-ray (EDX) analyses executed on a pristine sample [14].

Durapore^®^ polyvinylidene fluoride (PVDF) filter disks with a thickness of 125 µm and a pore size of 0.22 µm were purchased from Merck Millipore (Burlington, MA, USA).

Concentrated sulfuric acid (ACS reagent) with a purity of 95.0–97.0%, sodium chloride (ACS reagent) with a purity of ≥99.0% and sodium hydroxide pellets (ACS reagent) with a purity of ≥97.0% were acquired from Sigma-Aldrich Corporation.

The same supplier also provided Nafion^®^ 212 as a 30 cm × 30 cm polymeric sheet protected by two films that shielded the material from undesired contacts with the environment, so as to preserve its properties. It displayed a thickness of 50.8 µm and an equivalent weight of 2100 g eq^−1^.

### 2.2. Membranes Preparation

The manufacturing procedure of sulfonated graphene oxide samples initially involved a 15-min ultrasonication of 150 mL of the GO aqueous dispersion (600 mg of GO) placed in a round-bottomed flask, aimed at improving the overall homogeneity of the material. For this purpose, the Falc Instruments s.r.l. (Treviglio, Italy) ultrasound bath LABSONIC LBS 1-H3, ice-refrigerated to control the system temperature and, therefore, to avoid undesired overheating and thermal reduction, was used. After this step, an appropriate quantity of sulfuric acid was slowly incorporated in the flask, which was, subsequently, dipped into an oil bath and assembled with a reflux condenser and a magnetic stirrer. The functionalization process duration was six hours, the first three of which were carried out at ambient temperature (25 °C) and the remaining three at high temperature (100 °C). Magnetic mixing at 1500 rpm was applied during the entire functionalization timespan to guarantee a continuous homogenization of the dispersion. Dilution of the so-treated mixture, performed by means of 400 mL of deionized water, allowed an increase in pH up to values that could prevent problems during the subsequent filtration phase. In such step, the dispersion was vacuum-filtered on a PVDF filter disk placed on a Büchner funnel (14 cm in diameter) to drain the slurry and deposit the material. Additional 200 mL of deionized water was poured over the settling mixture in a controlled fashion in order to promote the removal of unreacted excess acid. In the end, the deposit was dried in an oven at 40 °C to favor the evaporation of water residues and conclude the self-assembly of the final product.

Six samples were prepared in this work by adopting different acid-to-GO molar ratios, namely, 1, 2.5, 5, 10, 15 and 20, a range that was identified as the most promising one in a previous work by the authors [14]. The GO molecular mass discussed in Section 2.1 was employed to evaluate the corresponding required acid volumes, as summarized in Table 1. The nomenclature chosen for the samples was SGO-X, in which X represented their specific acid-to-GO molar ratio.

A virgin GO sample was also prepared to be considered as a reference, starting from the same volume of 150 mL of commercial aqueous dispersion (600 mg of GO) ultrasonicated for 15 min in the presence of ice to mitigate the temperature increase. Vacuum-filtration and successive oven-drying at 40 °C allowed to complete the fabrication of the GO membrane.

### 2.3. Morphological and Microstructural Characterization

Thermogravimetric analysis (TGA) was executed through the Seiko Instruments Inc. EXSTAR 6000 TG/DTA 6300 by increasing the temperature from room conditions to 1000 °C via a heating ramp of 10 °C min^−1^. The chamber atmosphere was maintained inert by providing pure nitrogen at a flow rate of 55 mL min^−1^.

The ThermoElectron Continuμm IR microscope, combined with an FTIR Nicolet Nexus spectrometer, both provided by Thermo Fisher Scientific Inc. (Rodano, Italy), was used to acquire attenuated total reflection Fourier transform infrared (ATR-FTIR) spectra of the fabricated SGO-X membranes, along with those of benchmark GO, within a wavenumbers’ interval of 650–4000 cm^−1^. The experimental setup was created out of a single reflection silicon crystal and a mercury cadmium telluride (MCT) detector cooled by liquid nitrogen. A number of scans equal to 128 and a resolution of 4 cm^−1^ were applied during the execution of the tests.

Raman spectroscopy was performed by means of the Jobin Yvon LabRAM HR800 spectrometer by HORIBA (Kyoto, Kyoto), which was coupled with a 50x-objective Olympus BX41 microscope. The tests were conducted in a micro-Raman setup: the exciting source was a helium–neon (He–Ne) laser characterized by a wavelength of 632.8 nm, whose power was limited to 500 µW to minimize possible degradation and heating issues of the investigated materials.

X-ray diffraction (XRD) analysis was performed on all the as-prepared samples to infer information on their structural arrangement and stability as a function of temperature. Four subsequent conditions were implemented—30, 60, 120 °C and the cooled state—; the latter was reached after decreasing the specimens’ temperature from 120 °C to 30 °C. The temperature transitions were achieved at a heating rate of 10 °C min^−1^, followed by a 5-min holding before starting the measurements. The Rigaku SmartLab 9 kW, equipped with the Anton Paar DHS1100 high-temperature furnace and a D/teX Ultra 250 detector, was exploited for the described experiments. A cobalt (Co) filament was implemented as an X-ray source with a wavelength (λ) of 0.179 nm. An angular range (2θ) of 5–30° was covered during the tests with a step width of 0.02° and a scanning rate of 0.05° s^−1^. Interplanar distances d (nm) were calculated by means of Bragg’s law (Equation (1)) at the angle of incidence θ (°) associated with sharp reflections:

(1) $$d\;(\mathrm{nm})=\frac{\lambda }{2\sin\theta }$$

The Stereoscan 360 microscope, supplied by Cambridge Scientific Instrument Co. (Cambridge, UK), allowed the acquisition of scanning electron microscopy (SEM) images at 100× and 1000× magnifications. Accelerating voltage and current probe were set to 20 kV and 200 pA, respectively, and the chamber was operated under vacuum conditions. The apparatus employed for energy dispersive X-ray (EDX) spectroscopy experiments comprised of the scanning electron microscope model EVO 50 EP by Carl Zeiss AG and the INCA 200 PENTAFET LZ4 EDX spectrometer by Oxford Instruments plc. The accelerating voltage was 20 kV, the maximum current probe was 100 pA, and the pressure was kept in the range of 30–40 Pa. EDX measurements were carried out at 500× magnification on different zones of each sample; therefore, averaged compositional results were obtained.

### 2.4. Ion Exchange Capacity Evaluation

Ion exchange capacity (IEC) was assessed to define the role of sulfonic acid groups (–SO_3_H) in the ionic conduction mechanism of SGO-X samples by comparing their performance with the ones of pristine GO and Nafion^®^ 212. A classical acid–base titration method was executed, replicating a technique already exploited by the authors in previous works [14,22]. Firstly, the samples were dried in oven at 60 °C for 1 h and their dry mass was measured. Afterwards, they were equilibrated at room temperature in 250 mL of a 2 M sodium chloride (NaCl) aqueous solution for 48 h to allow the exchange of H^+^ ions, belonging to both oxygenated and sulfonated regions, with Na^+^ provided by the dissolved salt. After the equilibration time, the membrane portions were taken away and small volumes of a 0.01 M sodium hydroxide (NaOH) aqueous solution were used to titrate the original NaCl one. At each addition, the pH variation was recorded via the METTER TOLEDO^®^ MP220 Basic pH/mV/°C Meter in order to build the V_NaOH_–pH titration curves and determine the turning point values. IEC, which represents the milliequivalents of ionic sites containing exchangeable protons per gram of dried sample, was computed via Equation (2) as a function of the volume of NaOH solution V_NaOH_ (mL) required to reach the turning point, its concentration C_NaOH_ (mmol mL^−1^) and the dry mass of the samples m_dry_ (g):

(2) $$\mathrm{IEC}\;(\mathrm{meq}\;g^{-1})=\frac{V_{\mathrm{NaOH}}\;\cdot {\;C}_{\mathrm{NaOH}}}{m_{\mathrm{dry}}}$$

### 2.5. Proton Conductivity Investigation

The experimental setup exploited for the proton conductivity quantification via electrochemical impedance spectroscopy (EIS) comprised of a humid chamber designed to maintain the desired temperature and relative humidity (RH) levels for a total of ten combinations given by five different temperatures (20, 40, 60, 80, 100 °C) and two RH values (95% and 42%). Temperature was controlled by circulating heated oil through the outer jacket of the chamber and by inserting a thermocouple inside it. The thermohygrometer model C3121 by Comet System s.r.o. equipped with an external probe was employed to check the correct achievement of the RH values. The samples, previously trimmed in the shape of rectangles, underwent width and thickness measurements after being dried in an oven at 60 °C for 2 h. Then, they were clamped on a Teflon^®^ cell between two stainless-steel electrodes. Subsequently, the cell was inserted for 1 h in the jacketed chamber in order to subject the samples to the different temperature and relative humidity combinations. Afterwards, EIS analysis was conducted in a potentiostatic mode (frequency interval of 1–10^7^ Hz, amplitude of 0.5 V) through the Bode analyzer tool of the STEMlab^TM^ 125-14 board by Red Pitaya. Three measurements for each sample were surveyed to guarantee a certain reliability of the results. The collected Bode diagrams were firstly converted to the corresponding Nyquist plots and then fitted with the appropriate equivalent circuit in ZView^®^, a dedicated software by Scribner Associates Inc. Specifically, a modified Randles cell was chosen for this purpose [23]. It consisted of an overall ohmic resistance (R_s_) in series with an RC element. This element displays the internal resistance (R_i_) in parallel with a constant phase element (CPE) [23,24,25], which is the main difference between a modified and a regular Randles cell, the latter being characterized by an ideal capacitor. However, systems in which porosity and roughness of the electrode/electrolyte interface are relevant are intrinsically not ideal; hence, their equivalent circuit requires a CPE to properly describe the corresponding depressed Nyquist arcs. In a generic Nyquist plot, R_s_ corresponds to the intercept at high frequencies with the real impedance axis and accounts for ohmic losses, whereas the internal resistance of a material (R_i_) is represented by the diameter of the semicircle. Experimental values of R_i_ were extrapolated from the Nyquist plots and used to derive the proton conductivity σ (S cm^−1^) of the investigated membranes, expressed by the second Ohm’s law (Equation (3)):

(3) $${\sigma \;(S\;\mathrm{cm}}^{-1})=\frac{1}{\rho }=\frac{d}{R_{I}\;\cdot \;w\;\cdot \;t}$$

The involved quantities are the resistivity ρ (Ω cm), the spacing between the metal electrodes d (cm), the sample width w (cm) and the sample thickness t (cm).

## 3. Results and Discussion

### 3.1. Morphological and Microstructural Characterization

**Figure 1:**
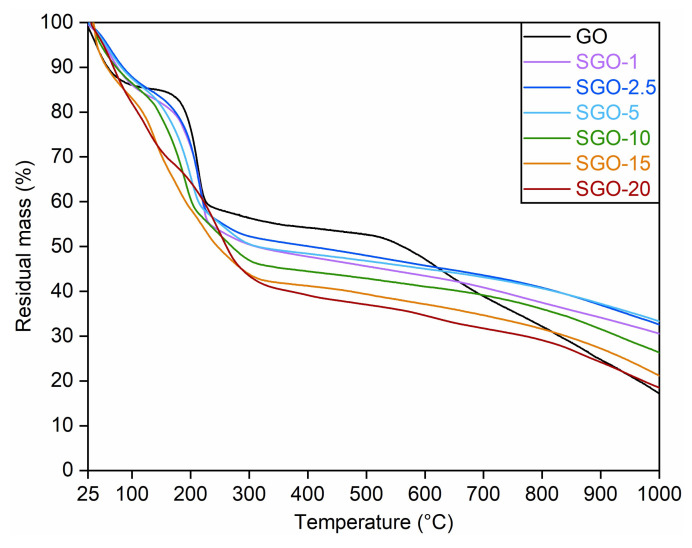
Thermograms of as-prepared SGO-X and benchmark GO membranes. Data of GO, SGO-1 and SGO-20 were reproduced with permission from [14].

The thermograms obtained from TGA are shown in Figure 1. Pristine GO was characterized by three main weight losses at an increasing temperature. The first one, which occurred below 100 °C and was about 15%, was associated with the removal of physically adsorbed water on the hydrophilic groups of GO planes [18,26]. The second one (about 25%) was detected close to 200 °C and may have referred to the decomposition of labile oxygenated functionalities (i.e., carboxyl, epoxide, hydroxyl and carbonyl groups), accompanied by the release of gaseous CO_x_ by-products [27,28]. The third step, resulting in a loss of approximately 35%, was instead recognized above 550 °C and could be related to the disruption of the graphitic planes of GO as a consequence of the high temperature level [18].

Concerning the sulfonated samples, SGO-1, SGO-2.5, SGO-5 and, partly, SGO-10, displayed very similar trends to that of the pure material. However, some slight differences could be observed. In particular, a more pronounced loss of oxygenated groups (almost 30%) was noticed at about 150 °C; therefore, at a temperature level lower than that of GO. In addition, a further weight reduction of nearly 6% was recorded above 250 °C, which was not perceptible in the virgin material. These two losses may be ascribed to the stability of the introduced sulfonic groups, which could vary depending on their position in the GO framework [29]. The moieties weakly bonded to the carbonaceous structure could probably be removed below 200 °C, whereas the ones able to form strong enough interactions may account for the second stage of mass drop. The latter was particularly pronounced (close to 11%) in SGO-10, which may suggest a more effective sulfonation with respect to membranes prepared with lower acid-to-GO molar ratios.

Contrarily, SGO-15 and SGO-20 exhibited a nearly continuous weight decrease in the 100–250 °C interval. The overlapping of the two previously discussed mass loss stages could be explained by an enhanced hydrophilic character of those samples, which potentially derived from a greater content of sulfonic groups and determined a larger amount of trapped water [18]. Moreover, their total mass loss in the investigated temperature range was higher compared to the other specimens, probably due to a reduced thermal stability caused by the high acid content. Hence, SGO-10 seemed to maintain the best thermal performance among the highly sulfonated samples, with a moderate mass loss in the temperature interval of interest for a possible fuel cell application and a residual mass larger than the benchmark GO.

**Figure 2:**
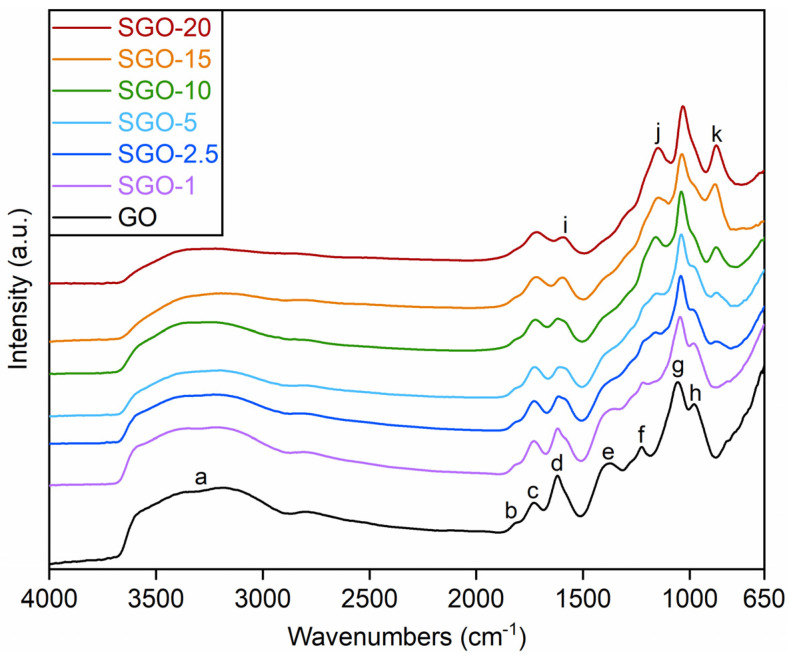
ATR-FTIR spectra of as-prepared SGO-X and benchmark GO membranes.

ATR-FTIR spectra of the SGO-X membranes are displayed in Figure 2, together with the reference GO; they were acquired in order to check differences in the distribution and intensity of the contributions of functional groups. Considering the extreme complexity of the investigated systems and that the structure of GO has not yet been unanimously established, the following analysis has to be intended as purely qualitative. Starting from GO, the broad band (a) between 3700 and 2800 cm^−1^ could be assigned to the stretching vibration of –OH groups into various functional groups [14,30]. Specifically, three primary contributions could be detected at 3700, 3400 and 3200 cm^−1^, referable to –OH groups into hydroxyl, carboxyl functionalities and adsorbed water, respectively [14]. Several GO characteristic bands could be recognized at lower wavenumbers. The small contribution detected at (b) 1818 cm^−1^ could be attributed to C=O stretching in anhydride groups. The one centered at (c) 1729 cm^−1^ could be associated with the stretching of C=O from carboxyl and carbonyl groups. The band centered at (d) 1619 cm^−1^ could be related to the O–H bending in adsorbed water [31]. The oneI) between 1400 and 1300 cm^−1^ could be assigned to the C–OH stretching in carboxyl groups and to the bending of O–H into hydroxyl, carboxyl and phenol moieties. The contribution (f) between 1300 and 1200 cm^−1^ could mainly refer to the stretching vibration of –COC– from epoxide groups and, secondly, to other moieties such as ethers, esters and phenol ones [31,32]. Finally, two other bands could be identified at about (g) 1051 cm^−1^ and (h) 981 cm^−1^. The former could be related to the stretching of C–OH bonds in hydroxyl moieties, whereas the latter could be attributed to the stretching of C–O from unstable groups, typically lactols and peroxides [14].

SGO-X spectra exhibited some dissimilarities from the one of pristine GO. The contribution at (i) 1580 cm^−1^, which could be attributed to the stretching vibration of C=C bonds in sp^2^-hybridized regions [32,33], seemed to progressively increase in intensity, with respect to the adjacent (d) band, while raising the acid-to-GO molar ratio. A possible explanation is the interaction of sulfuric acid with weaker oxygenated functionalities, which were removed with a consequent partial restoration of graphitic domains. Such a phenomenon appeared to be accompanied by the partial loss of hydroxyl groups, whose contribution at (a) 3700–3400 cm^−1^ was less intense. Reynosa-Martínez et al. [34] proposed a leaching effect as the main mechanism for the re-establishment of graphitic double bonds due to the action of sulfuric acid, mainly involving the C–OH groups on the basal planes of GO. Conversely, C=O and –COOH functionalities were reported as difficult to reduce, given the high activation energy of the corresponding reactions. Other variations from pure GO were detected at wavenumbers lower than 1500 cm^−1^. A small rise in the band centered roughly at (j) 1150 cm^−1^ could be observed. Such a trend could be partially ascribed to the stretching of the O=S=O bonds in sulfonic groups (–SO_3_H) [14,35], albeit other functionalities could influence its intensity and width. Similarly, the band arising at (k) 880–870 cm^−1^ could be related to the stretching of S–O in sulfinic (–SO_2_H) and sulfonic groups [14,36]. A growth in the intensity of these bands with respect to the adjacent (g) mode could be appreciated at higher acid-to-GO molar ratios, suggesting a higher functionalization degree fostered by a larger sulfuric acid content.

In parallel to these observations, one could notice the attenuation of baI (e) 1300–1400 cm^−1^ and (h), about 981 cm^−1^, which were recorded for pure GO. This phenomenon could be explained by assuming the removal of a fraction of the less stable lactol, peroxide and carboxyl groups due to the sulfonation process. Such a result may once again indicate the possible reduction caused by the acid when added in large quantities.

**Figure 3:**
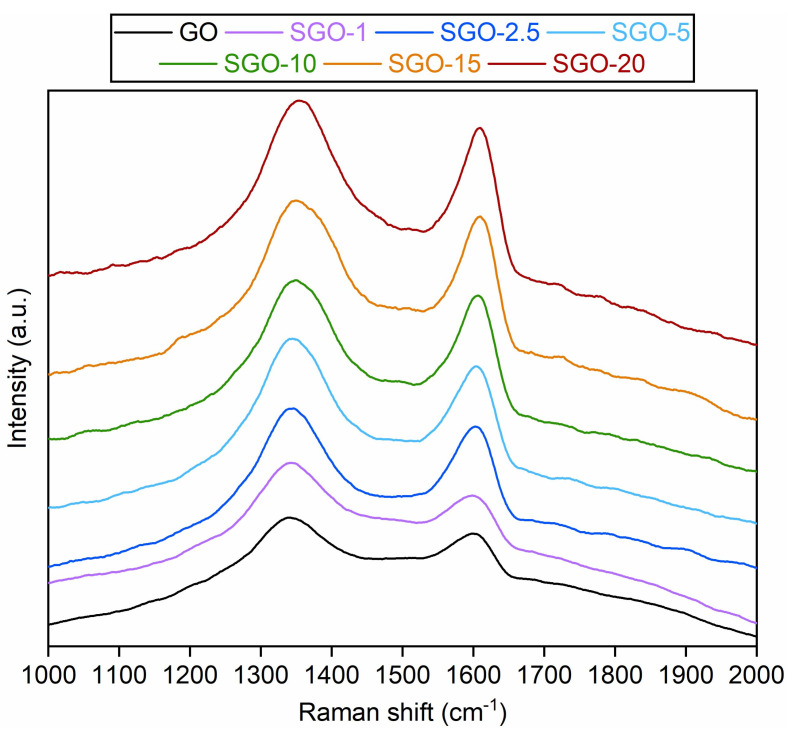
Raman spectra of the as-prepared SGO-X and benchmark GO membranes.

Raman spectra of the SGO-X samples and benchmark GO were collected in Figure 3. All spectra were characterized by two clearly recognizable bands. The first one, centered at about 1350 cm^−1^, is commonly referred to as the D band and derives from the structural disorder of the material. This vibrational mode was virtually absent in the crystalline graphite, which showed a high degree of order fostered by the sp^2^-hybridized carbons. On the contrary, GO-based materials possessed impurities due to the oxidation process, and so regions rich in carbons covalently bonded with O-bearing groups, which were generally assessed as the main contributions to the prominent D band [2,37]. The second band, located roughly at 1600 cm^−1^, is indicated as the G band [38]. It is related to the presence of graphitic domains within the GO structure [39] and its intensity grows with higher concentrations of conjugated and doubly bonded carbon atoms. A slight fading and broadening of the G band could be noticed moving from the virgin GO to the sulfonated samples, probably related to a partial amorphization of GO due to the interaction with the sulfonating species.

The ratio between the D and G band intensities (I_D_/I_G_) could be deemed as a good parameter to estimate the degree of defectiveness in GO layers [2], albeit an interdependence of the I_D_/I_G_ values and the excitation laser energy should be considered [37]. The I_D_/I_G_ results for the investigated samples are reported in Table 2. The values seemed to lower with the increase in the acid-to-GO molar ratio. Such a trend could again hint the expansion of graphite-like zones in the membranes prepared with a high sulfuric acid content, as a consequence of the previously mentioned leaching effect. The minimum I_D_/I_G_ value, registered for SGO-15, could indicate that an acid-to-GO ratio greater than 10 started to have a detrimental effect on the sp^3^-hybridized carbon framework, leading to a partial reconstitution of sp^2^ domains. On the contrary, the higher result derived for SGO-20 could imply that functionalization issues occurred at the greatest sulfuric acid loading.

**Figure 4:**
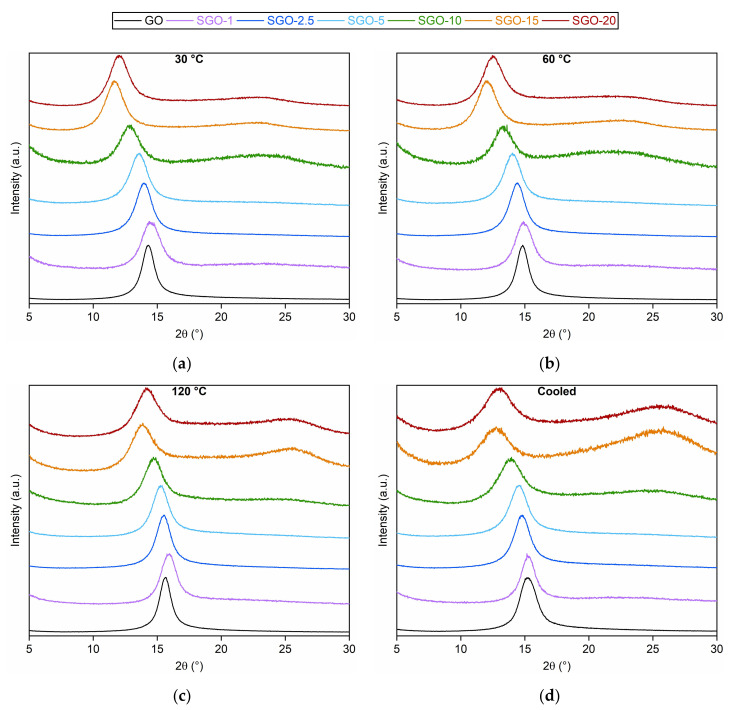
Diffractograms of the as-prepared SGO-X and benchmark GO membranes at (**a**) 30 °C, (**b**) 60 °C, (**c**) 120 °C and (**d**) cooled state.

The diffractograms acquired for the analyzed SGO-X and reference GO membranes at 30, 60, 120 °C and in the cooled state are illustrated in Figure 4. The corresponding interlayer distances, derived from Equation (1), are listed in Table 3. Starting from the results obtained at 30 °C (Figure 4a), a well-defined reflection was detected for all the specimens at a diffraction angle between 11.5° and 14.5°. Such a range was much different from that of graphite (22–23°), inasmuch the presence of oxygenated functionalities in the GO framework induced a larger distance between adjacent layers [2,40,41]. Except for SGO-1, whose sulfuric acid content was not sufficient to provoke a deviation from GO, the increase in the acid-to-GO molar ratio involved reflections shifted to lower diffraction angles that were equivalent to higher interlayer distances. This trend could be interpreted as a consequence of the presence of sulfonic acid groups [17,41]. Nonetheless, SGO-20 proved a lowered interplanar distance with respect to SGO-15 (0.85 nm and 0.88 nm, respectively). Such an outcome could be attributed to a lower functionalization efficiency and, therefore, to less –SO_3_H groups introduced on GO basal planes.

The characteristic GO reflection moved towards higher diffraction angles with the increase in the experimental temperature (range 12–15° at 60 °C in Figure 4b, range 13.5–16° at 120 °C in Figure 4c), leading to lower interlayer spacing for all the examined membranes. A slight thermal reduction with the loss of unstable O-bearing functional groups could be hypothesized. Hydroxyl groups with a low binding energy have been reported to dissociate even at room temperature [27,33], while some carboxyl moieties are supposed to be removed at about 100–150 °C [42]. This phenomenon could be seen as a sort of preliminary step of the mass loss measured approaching 200 °C during thermogravimetric analyses (Figure 1). The cooled condition (Figure 4d) appeared to strengthen the previous interpretation, since the related interplanar distance values were intermediate with respect to those at 60 °C and 120 °C. On the one hand, the larger interplanar distance in comparison to the ones extrapolated at 120 °C was probably caused by the re-adsorption of humidity within GO layers. On the other, the lower values in comparison to the ones observed at 60 °C and 30 °C could confirm the slight thermal reduction in the studied materials.

Another evidence of the modifications having occurred at 120 °C could be the rise in the flat and broad reflection near 22–27° in the diffractograms of SGO-15 and SGO-20. This interval was similar to the typical diffraction angle range of crystalline graphite, equivalent to a stacking distance of about 0.34 nm [2,43]. The reflection was even more evident in the cooled state, as if high-temperature levels induced permanent structural alterations on these samples. As already deduced from ATR-FTIR (Figure 2) and Raman (Figure 3) spectra, the membranes characterized by high acid loadings could already be prone to a transition towards a graphitic structure; ergo, the thermal effect could be facilitated.

**Figure 5:**
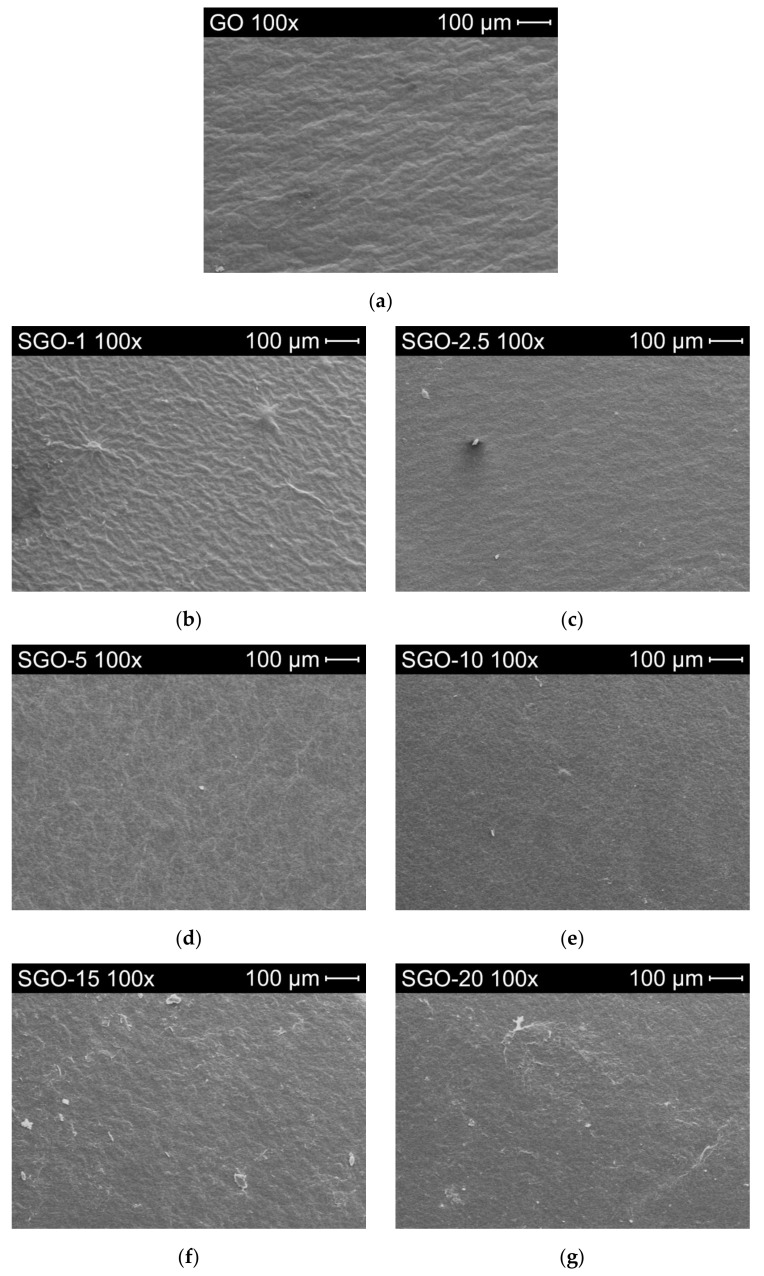
SEM images at 100× magnification of: (**a**) pure GO; (**b**) SGO-1; (**c**) SGO-2.5; (**d**) SGO-5; (**e**) SGO-10; (**f**) SGO-15; (**g**) SGO-20.

Figure 5 and Figure S1 collect the surface images captured through scanning electron microscopy for all the as-prepared samples. Two different magnifications were analyzed, i.e., 100× (Figure 5) and 1000× (Figure S1), so as to obtain a more detailed view of possible morphological changes resulting from the functionalization process. The surfaces of SGO-X samples were rather homogenous altogether and, compared to pristine GO (Figure 5a), they did not show significant modifications brought by the addition of sulfuric acid. Regardless, they appeared slightly more wrinkled with respect to the smoother surface of GO, even in the case of SGO-1 (Figure 5b). This characteristic could confirm the occurrence of some morphological alteration even at the lowest acid-to-GO molar ratio. According to Reynosa-Martínez et al. [34], wrinkles can be generated by an increase in the number of oxygenated functionalities, which induce tensions in the network. Therefore, the undulation arising on the SGO-X surfaces could have perhaps been provoked by the interaction with sulfuric acid that enriched pure GO with further O-bearing moieties. Whether they simply were –SO_3_H groups or a chemical transition from sp^2^- to sp^3^-hybridized regions with the formation of new C–O bonds [44] is yet to be cleared up.

**Figure 6:**
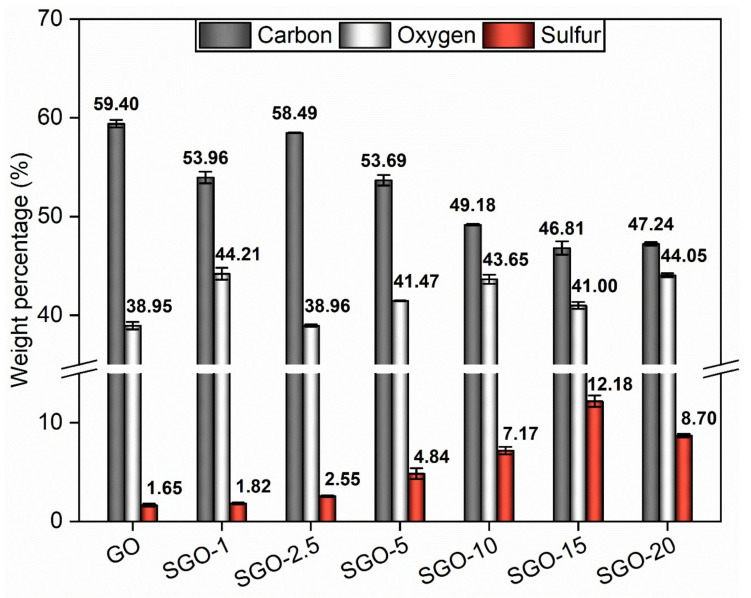
EDX spectroscopy results of the as-prepared SGO-X and benchmark GO membranes.

The semi-quantitative elemental data derived from EDX spectra are summarized in Figure 6. The benchmark GO carbon-to-oxygen atomic percentage ratio (2.03 ± 0.03) appeared to be consistent with the typical one indicated in the literature (1.5–2.5) [45]. The 1.65 wt.% of sulfur (S) recorded for the virgin material had to be ascribed to the use of sulfur-containing species in the synthesis process [19,46]. As expected, the proposed functionalization method affected the composition of the GO framework. A reduction in the weight percentage of carbon could be observed, as well as a rise in sulfur and oxygen contents even at the lowest sulfonation degree (SGO-1). Such an outcome suggested a successful introduction of –SO_3_H groups by means of sulfuric acid.

Nevertheless, SGO-2.5, SGO-15 and SGO-20 elemental analyses allowed to determine some considerations. First of all, the manufacturing stages should still be optimized, inasmuch SGO-2.5 appeared more analogous to GO than to SGO-1, in terms of the C/O ratio. Regarding SGO-15, the amount of employed acid probably surpassed a threshold value over which a partial reduction in GO layers was promoted, as previously discussed. Consequently, some oxygenated groups were likely to be removed and the overall oxygen content diminished. Such a result agreed with the ATR-FTIR spectrum (Figure 2) of this sample, as well as with its I_D_/I_G_ ratio (Table 2), which turned out to be the lowest; thus, this indicated the partial restoration of ordered graphitic domains. A further increase in the acid-to-GO ratio (SGO-20) led to a growth in the oxygen weight percentage and a decrease in that of sulfur, approaching values that were comparable to those of SGO-10. One might attribute this outcome to a less effective functionalization, which caused fewer sulfonic acid groups to be introduced on the GO planes. The I_D_/I_G_ ratio derived for SGO-20 (1.29 ± 0.13) and the analysis of XRD patterns (Figure 4) could support this interpretation, since the pre-existing sp^3^-hybridized carbons seemed to remain dominant with respect to the sp^2^-hybridized ones, similarly to pristine GO [47].

### 3.2. Ion Exchange Capacity

**Figure 7:**
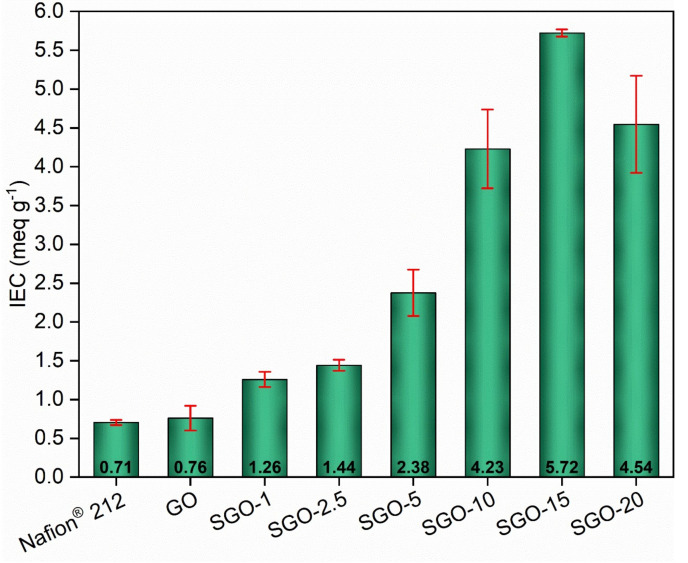
IEC values of the as-prepared SGO-X membranes compared to benchmark GO and Nafion^®^ 212.

The IEC was determined with the purpose of understanding how the presence of sulfonic acid groups influenced the ability of GO to carry ions. The results, obtained via Equation (2) after the titration procedure explained in Section 2.4, are shown in Figure 7. All the fabricated SGO-X membranes outperformed both Nafion^®^ 212 and pristine GO. A value of 0.71 ± 0.03 meq g^−1^ was measured for benchmark Nafion^®^ 212. It was slightly lower than the one reported in the literature, which was about 0.90 meq g^−1^ [48,49,50]. Such an outcome may be the consequence of the preliminary treatment in the oven at 60 °C, which could have damaged the most superficial –SO_3_H groups. Nevertheless, one should consider that the drying phase was crucial to remove traces of water trapped in the material and obtain reliable measurements of the dry weight. The recorded value for pure GO (0.76 ± 0.16 meq g^−1^) was practically equivalent to that of Nafion^®^ 212 and did not excessively differ from the values surveyed in a preceding work by the authors [14] and found in the literature [1,12].

The sulfonated membranes demonstrated an upward IEC tendency parallel to the increase in the acid-to-GO molar ratio. SGO-1, whose IEC value was 1.26 ± 0.10 meq g^−1^, disclosed an almost two-fold improvement with respect to GO. Once again, an actual modification of the original structure was indicated, even at the minimum sulfuric acid amount. SGO-2.5 was comparable with SGO-1, whereas large improvements were recorded for SGO-5 (2.38 ± 0.30 meq g^−1^), SGO-10 (4.23 ± 0.51 meq g^−1^) and SGO-15 (5.72 ± 0.05 meq g^−1^). SGO-15 exhibited the highest IEC among all tested samples, with an eight-fold enhancement over pure GO. The additional increase in the acid-to-GO molar ratio up to 20 in SGO-20 (4.54 ± 0.63 meq g^−1^) did not guarantee comparable IEC values, but the result was still noteworthy despite the previously hypothesized less effective functionalization. Such considerations were reinforced by the EDX elemental analysis (Figure 6), since the evolution of the IEC performance appeared to reflect the trend of the measured sulfur content, which could be associated, by analogy, with the amount of sulfonic acid groups effectively inserted on the GO layers.

### 3.3. Proton Conductivity

**Figure 8:**
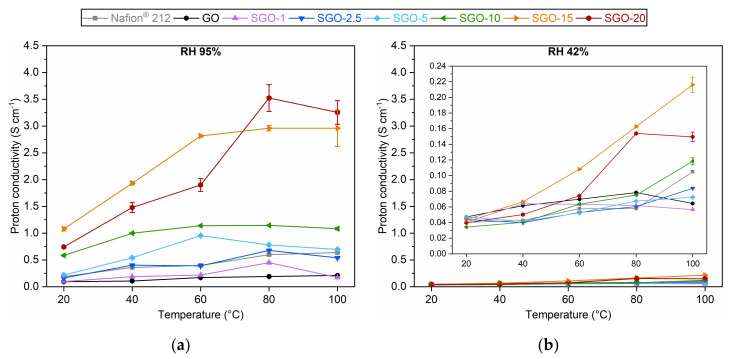
Proton conductivity values at (**a**) 95% RH and (**b**) 42% RH of the as-prepared SGO-X membranes compared to the benchmark GO and Nafion^®^ 212 in the interval of 20–100 °C.

Electrochemical impedance spectroscopy permitted the derivation of proton conductivity values for all the as-prepared SGO-X samples, as well as for the reference Nafion^®^ 212 and pure GO. The results obtained at both high (95%) and medium (42%) RH are displayed in Figure 8. In general, proton conductivity increased with temperature. This trend was expected, since the energy barrier to overcome to trigger protons’ mobility was more easily surpassed at higher temperatures [40]. The SGO-X membranes produced the best performances at high RH (Figure 8a), thanks to the influence of polar S-bearing groups that were able to gather a considerable amount of water and to form ionic clusters that encouraged proton diffusion [35]. The only exception was SGO-1, whose low functionalization degree prevented a clear improvement with respect to GO. The virgin material showed the overall worst results, since its oxygenated functionalities could have been insufficient to guarantee an adequate proton conductivity [1], despite the good water sorption ability demonstrated from the water uptake (WU) tests (Section S2). The SGO-2.5 results were superimposable to the ones of Nafion^®^ 212. One should notice that the extrapolated values for the commercial material were larger than the typical results reported in the literature, which were about 0.1 S cm^−1^ [1,51,52,53]. Such an inconsistency could be ascribed to the condensation of water droplets on the surface of the stainless-steel electrodes during the tests, which could have altered the final measurements. Nonetheless, the overall trend could still be considered reliable, given that the experiments were conducted on all membrane samples with the same test conditions. A further increase in the acid-to-GO molar ratio up to 15 was accompanied by a continuous improvement of the proton conductivity up to values of 1–3 S cm^−1^. Then, SGO-20 displayed slightly worse results in the 20–60 °C interval. This behavior strongly agreed with the previously discussed EDX (Figure 6) and IEC (Figure 7) outcomes, as well as with those of WU tests (Section S2). The SGO-10 trend was the most consistent among all investigated specimens, with a proton conductivity surpassing 1 S cm^−1^. Moreover, the restrained values’ variability could hint the good homogeneity of this sample. It is necessary to specify that the sulfonated membranes exhibited substantially higher outcomes than the ones reported in the literature for innovative proton conductors [13,51,52,54,55]. A possible explanation is the presence of small traces of sulfuric acid trapped in between the stacked GO layers, which could have led to an overestimation of the real conductivity value.

The considerations determined for high RH results could be extended to medium RH ones as well (Figure 8b), albeit the proton conductivity was drastically reduced due to the partial inhibition of the water-mediated conduction mechanism [40]. In fact, it is believed that the Grotthuss mechanism dominates when the availability of hydrated pathways is reduced [18]. Differently from the high RH case, pure GO proved a better performance with respect to Nafion^®^ 212, in accordance with the higher WU values observed in medium humidification conditions (Section S2). The samples characterized by lower sulfonation degrees (SGO-1, SGO-2.5, SGO-5) demonstrated quite similar proton conductivity values, following the observations acquired from the water uptake evaluation. A substantial enhancement was detected for SGO-10, especially at high temperatures. In such conditions, it outperformed both Nafion^®^ 212 and pristine GO with a proton conductivity of 0.118 ± 0.005 S cm^−1^. An additional performance growth was registered for SGO-15, whereas SGO-20 was not able to reach the same outcomes, most likely because of the speculated loss in the sulfonation effectiveness.

## 4. Conclusions

Self-standing sulfonated graphene oxide (SGO-X) membranes were prepared from virgin GO through a simple and easily reproducible method relying on the vacuum filtration of sulfuric acid-modified dispersions and low-temperature oven-drying of the deposits. Six acid-to-GO molar ratios were investigated to study different functionalization degrees.

The extensive morphological and microstructural characterization performed on the samples suggested the effectiveness of the proposed procedure. The actual modification of the GO structure with the introduction of sulfonic acid groups was supposed even at low acid loadings from the analysis of ATR-FTIR spectra, XRD patterns and EDX compositional results. The infrared spectroscopy allowed us to detect different bands in SGO-X samples with respect to pure GO, which were attributed to the stretching vibrations of sulfur-bearing moieties. The interplanar distance derived from diffractograms grew with the acid-to-GO molar ratio, due to the presence of sulfonic acid groups. The sulfur content in the SGO-X samples underwent a continuous growth parallel to the employed sulfuric acid amount up to SGO-15.

Several evidences of loss in the functionalization efficiency were detected at an acid-to-GO molar ratio of 20 by Raman spectroscopy, EDX measurements and XRD analysis, which suggested a smaller quantity of –SO_3_H groups introduced on GO planes and the partial restoration of graphitic domains, as also observed from the more intense stretching vibration of C=C bonds in ATR-FTIR spectra.

The functional characterization of the SGO-X samples allowed a comparison with pure GO and commercial Nafion^®^ 212. The sulfonated materials demonstrated an improved ion exchange capacity, up to values ranging from 4 to 5 meq g^−1^ for SGO-10, SGO-15 and SGO-20. Proton conductivity values were extrapolated from EIS measurements. The trend with respect to temperature and relative humidity agreed with the results obtained from the IEC tests. The samples characterized by a high acid-to-GO molar ratio provided the best outcomes at both high and medium RH.

In light of the results obtained, SGO-10 can be regarded as the most promising GO-derived material among the presented ones. It proved a less widespread presence of graphite-like domains, an adequate homogeneity and a high ion exchange capacity of 4.23 ± 0.51 meq g^−1^. Furthermore, it displayed a stable proton conductivity at high humidity and a better value than GO and Nafion^®^ 212 at 100 °C and 42% RH. Additional research should be devoted to more thoroughly investigating the effects of the proposed functionalization procedure on both the structure and properties of the membranes, by means of additional morphological (e.g., X-ray photoelectron spectroscopy) and functional (e.g., tensile strength tests) analyses. The aim is to optimize both the composition and structural stability of these materials, in order to achieve a successful characterization of their in situ behavior in a functioning fuel cell.

## Figures and Tables

**Table 1:** Sulfuric acid quantities used to produce the six SGO-X samples.

Sample	Acid-to-GO Molar Ratio	Moles of Acid (mol)	Volume of Acid (mL)
SGO-1	1	0.017	0.90
SGO-2.5	2.5	0.042	2.25
SGO-5	5	0.085	4.50
SGO-10	10	0.170	9.00
SGO-15	15	0.255	13.50
SGO-20	20	0.340	18.00

**Table 2:** Raman band positions and intensity ratios of the as-prepared SGO-X samples and benchmark GO.

Sample	D Band Position (cm^−1^)	G Band Position (cm^−1^)	D/G Intensity Ratio
GO	1340	1599	1.53 ± 0.06
SGO-1	1344	1598	1.60 ± 0.03
SGO-2.5	1347	1602	1.36 ± 0.08
SGO-5	1345	1604	1.41 ± 0.02
SGO-10	1350	1606	1.08 ± 0.01
SGO-15	1349	1609	0.98 ± 0.03
SGO-20	1353	1609	1.29 ± 0.13

**Table 3:** Interplanar distance of the as-prepared SGO-X and benchmark GO membranes at different temperatures, computed via Bragg’s law.

Sample	Temperature (°C)			
	30	60	120	30 (Cooled)
	Interplanar Distance (nm)			
GO	0.72	0.69	0.66	0.68
SGO-1	0.71	0.69	0.65	0.68
SGO-2.5	0.74	0.71	0.67	0.70
SGO-5	0.75	0.73	0.68	0.71
SGO-10	0.79	0.76	0.70	0.74
SGO-15	0.88	0.85	0.74	0.81
SGO-20	0.85	0.83	0.73	0.78

## Data Availability

Not applicable.

## Supplementary Materials

The following supporting information can be downloaded, Section S1: Scanning electron microscopy, Section S2: Assessment of water uptake, Table S1: Water uptake results at 95% RH of the as-prepared SGO-X membranes compared to reference GO and Nafion^®^ 212, Table S2: Water uptake results at 42% RH of the as-prepared SGO-X membranes compared to reference GO and Nafion^®^ 212. Reference [56] is cited in Supplementary Materials.

## Sample Availability

Samples of the compounds are available from the authors.
