# Supplementary material for: Investigation of Sulfonated Graphene Oxide as the Base Material for Novel Proton Exchange Membranes
Source: Molecules. 2022 Feb 23;27(5):1507. doi: 10.3390/molecules27051507 (PMC8912047; doi:10.3390/molecules27051507)
Supplement: Supplementary file 1 [file molecules-27-01507-s001.zip › molecules-1601816-supplementary.pdf]

Supplementary Information

Investigation of sulfonated graphene oxide as the base material for novel proton exchange membranes

Andrea Basso Peressut [1], Matteo Di Virgilio [1,*], Antonella Bombino [1], Saverio Latorrata [1], Esa Muurinen [2], Riitta L. Keiski [2] and Giovanni Dotelli [1,*]

[1] Department of Chemistry, Materials and Chemical Engineering "Giulio Natta", Politecnico di Milano, Piazza Leonardo da Vinci 32, 20133 Milano, Italy;

[2] Environmental and Chemical Engineering Research Unit, Faculty of Technology, University of Oulu, Pentti Kaiteran katu 1, FI-90014 Oulu, Finland

* Correspondence: matteo.divirgilio@polimi.it; giovanni.dotelli@polimi.it

S1. Scanning electron microscopy

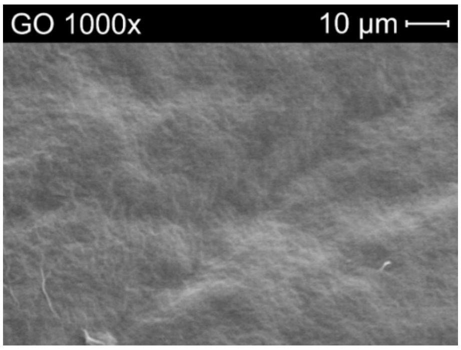

(**a**)

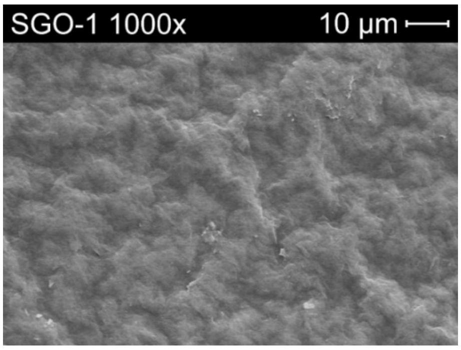

(**b**)

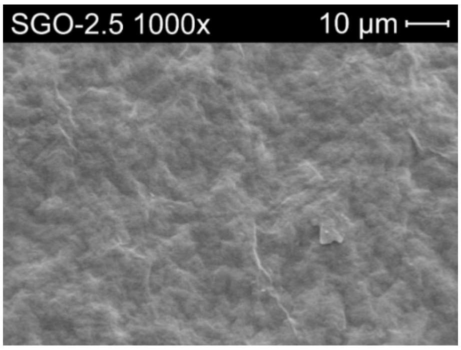

(**c**)

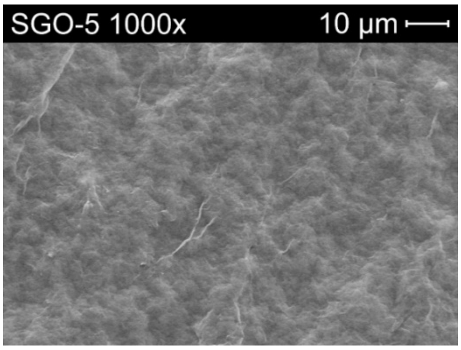

(**d**)

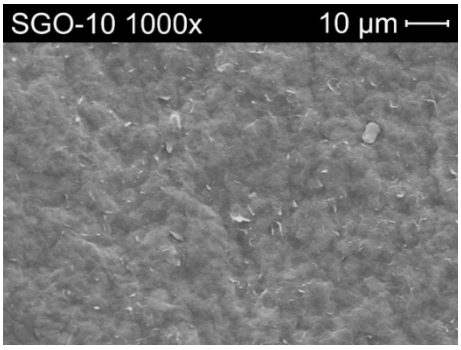

(**e**)

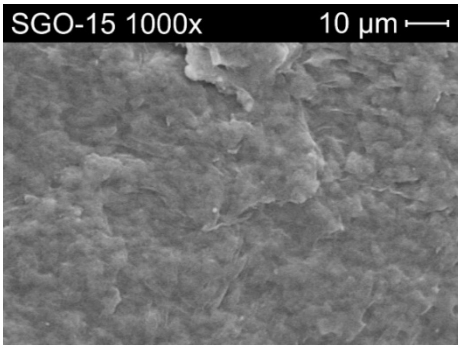

(**f**)

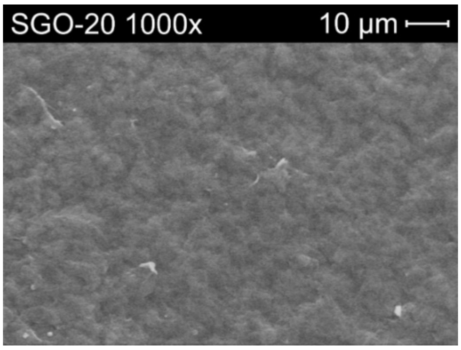

(**g**)

Figure S1. SEM images at 1000x magnification of (**a**) pure GO; (**b**) SGO-1; (**c**) SGO-2.5; (**d**) SGO-5; (**e**) SGO-10; (**f**) SGO-15; (**g**) SGO-20.

S2. Assessment of water uptake

Water uptake (WU), defined as the percentage increment of a membrane mass as a result of water adsorption, can be expressed by means of Equation S1, in which m_{wet} (g) and m_{dry} (g) are the wet and dry mass of the sample, respectively:

$$WU\ (\%) = \frac{m_{wet} - m_{dry}}{m_{dry}} \cdot 100 \qquad (S1)$$

The investigation of this property for SGO-X, benchmark GO and Nafion® 212 membranes, cropped in a rectangular shape, has initially required an oven-drying step at 60 °C for 2 hours, with the purpose of removing possible traces of residual moisture. Then, the dry mass (m_{dry}) has been immediately recorded by using an analytical balance. Subsequently, the dry specimens have been hanged on a Teflon®-coated stainless-steel sample holder, which has been positioned into the same humid chamber employed for proton conductivity tests, as described in Section 2.5. The same testing conditions have been applied as well, with the temperature varying in the 20–100 °C range and the relative humidity set at either 95% or 42%. After 1 hour inside the humid environment, the samples have been removed from the holder and cleared from superficial water, then the wet mass (m_{wet}) has been recorded.

The results obtained at high RH (95%) are reported in Table S1. The SGO-X samples can be divided in two groups depending on their response to the humid environment. The first group includes SGO-1, SGO-2.5 and SGO-5, characterized by low acid contents. These specimens prove, on average, an improved water retention performance with respect to both Nafion® 212 and GO. Concerning the benchmark materials, the former exhibits a fairly constant water uptake over the whole temperature range. However, it seems to suffer a slight water sorption drop at the highest temperatures (80-100 °C), probably related to a slight thermally induced dehydration, favored by the presence of the hydrophobic PTFE-based chain. An almost constant WU trend is recognized for the latter as well, albeit it is established at greater values with respect to Nafion® 212. This outcome underlines the good hydrophilic nature conferred by the oxygenated moieties that decorate GO structure, which is further reinforced by the introduction of sulfonic acid groups in the SGO-X samples. Nonetheless, a WU decrease is registered at the highest temperatures for SGO-1, SGO-2.5 and SGO-5, probably related to a shortage of sulfonated substituents, which impedes to maintain a substantial hydration. The second group includes SGO-10, SGO-15 and SGO-20, with higher acid-to-GO molar ratios. For these samples, the overall improvement in the water retention capability is clear with respect to the membranes belonging to the first group. Therefore, WU appears to keep enhancing with the increase in the sulfuric acid content, as a consequence of the larger quantity of hydrophilic functionalities inserted in the GO framework. SGO-10 reveals a rather stable behavior, differently from SGO-15 and SGO-20 that display a much more variable performance in the investigated temperature range. In the case of SGO-15, the main causes could be the

defectiveness of its structure and the presence of random reduced domains, as suggested by ATR-FTIR (Figure 2) and Raman (Figure 3) spectra. Regarding SGO-20, such trend could be attributed to a flawed functionalization, as also emerged from XRD patterns (Figure 4) and EDX analysis (Figure 6).

The WU values measured at medium RH (42%) are highlighted in Table S2. A global decrease in the amount of retained water is outlined for all the analyzed samples. The performance worsening is particularly evident for Nafion® 212, especially at 100 °C due to the strong decrease in mechanical resistance as a consequence of its low glass transition temperature [56]. GO hydrophilicity limits its dehydration, therefore determining larger water uptake even at the highest temperatures. The discrepancies among the SGO-X membranes are considerably narrowed, since their trends are comparable on the entire temperature interval. The introduction of $-SO_3H$ groups bestows a further improvement of water sorption with respect to the virgin GO. Among the tested materials, SGO-5, SGO-10 and SGO-15 proved to be the most promising, with a fairly constant trend and the best WU outcomes at 80 and 100 °C. The existence of a threshold acid-to-GO molar ratio over which the functionalization is less effective is again suggested, inasmuch SGO-20 undergoes a water uptake deterioration in a fashion similar to the one noticed at high relative humidity.

Table S1. Water uptake results at 95% RH of the as-prepared SGO-X membranes compared to reference GO and Nafion® 212.

Sample	Temperature (°C)				
	20	40	60	80	100
	Water uptake (%)				
Nafion® 212	6.89 ± 0.74	11.16 ± 2.17	9.18 ± 4.82	6.42 ± 1.38	6.16 ± 1.32
GO	16.91 ± 0.75	24.25 ± 4.88	18.73 ± 0.83	18.12 ± 2.44	10.56 ± 1.78
SGO-1	34.72 ± 5.18	34.94 ± 8.55	30.46 ± 9.84	13.58 ± 1.23	14.80 ± 3.55
SGO-2.5	33.37 ± 5.88	35.55 ± 2.95	28.70 ± 4.90	13.10 ± 0.72	10.19 ± 0.07
SGO-5	31.36 ± 3.49	48.66 ± 0.33	39.93 ± 10.20	22.10 ± 0.41	20.62 ± 1.45
SGO-10	53.27 ± 1.65	71.65 ± 10.32	70.50 ± 5.67	45.08 ± 5.75	51.70 ± 1.63
SGO-15	64.75 ± 4.20	79.22 ± 23.43	71.45 ± 2.21	50.71 ± 0.59	49.27 ± 0.67
SGO-20	65.15 ± 3.38	67.68 ± 8.60	68.75 ± 6.78	65.93 ± 1.69	44.60 ± 9.80

Table S2. Water uptake results at 42% RH of the as-prepared SGO-X membranes compared to reference GO and Nafion® 212.

Sample	Temperature (°C)				
	20	40	60	80	100
	Water uptake (%)				
Nafion® 212	2.00 ± 0.52	3.78 ± 0.23	3.38 ± 0.40	2.84 ± 0.33	1.76 ± 0.47
GO	3.43 ± 1.20	8.82 ± 1.67	11.57 ± 0.67	8.89 ± 0.04	6.53 ± 0.26
SGO-1	4.94 ± 1.21	8.77 ± 0.69	12.45 ± 2.82	10.56 ± 1.05	9.44 ± 1.55
SGO-2.5	6.15 ± 0.29	7.30 ± 1.21	9.74 ± 0.24	10.25 ± 3.33	6.96 ± 1.44
SGO-5	7.56 ± 0.98	7.15 ± 0.77	12.81 ± 1.91	11.27 ± 0.95	9.10 ± 0.95
SGO-10	11.33 ± 1.99	14.39 ± 2.13	13.91 ± 2.24	13.00 ± 1.78	12.12 ± 2.21
SGO-15	9.90 ± 0.45	9.53 ± 0.15	12.47 ± 1.35	8.94 ± 0.71	9.98 ± 0.21
SGO-20	12.20 ± 1.56	13.63 ± 0.73	15.38 ± 2.64	9.38 ± 0.88	9.43 ± 1.53
